# Persistent neurologic symptoms and cognitive dysfunction in non‐hospitalized Covid‐19 “long haulers”

**DOI:** 10.1002/acn3.51350

**Published:** 2021-03-30

**Authors:** Edith L. Graham, Jeffrey R. Clark, Zachary S. Orban, Patrick H. Lim, April L. Szymanski, Carolyn Taylor, Rebecca M. DiBiase, Dan Tong Jia, Roumen Balabanov, Sam U. Ho, Ayush Batra, Eric M. Liotta, Igor J. Koralnik

**Affiliations:** ^1^ Davee Department of Neurology Northwestern University Feinberg School of Medicine Chicago Illinois USA

## Abstract

**Objective:**

Most SARS‐CoV‐2‐infected individuals never require hospitalization. However, some develop prolonged symptoms. We sought to characterize the spectrum of neurologic manifestations in non‐hospitalized Covid‐19 “long haulers”.

**Methods:**

This is a prospective study of the first 100 consecutive patients (50 SARS‐CoV‐2 laboratory‐positive (SARS‐CoV‐2^+^) and 50 laboratory‐negative (SARS‐CoV‐2^‐^) individuals) presenting to our Neuro‐Covid‐19 clinic between May and November 2020. Due to early pandemic testing limitations, patients were included if they met Infectious Diseases Society of America symptoms of Covid‐19, were never hospitalized for pneumonia or hypoxemia, and had neurologic symptoms lasting over 6 weeks. We recorded the frequency of neurologic symptoms and analyzed patient‐reported quality of life measures and standardized cognitive assessments.

**Results:**

Mean age was 43.2 ± 11.3 years, 70% were female, and 48% were evaluated in televisits. The most frequent comorbidities were depression/anxiety (42%) and autoimmune disease (16%). The main neurologic manifestations were: “brain fog” (81%), headache (68%), numbness/tingling (60%), dysgeusia (59%), anosmia (55%), and myalgias (55%), with only anosmia being more frequent in SARS‐CoV‐2^+^ than SARS‐CoV‐2^‐^ patients (37/50 [74%] vs. 18/50 [36%]; *p* < 0.001). Moreover, 85% also experienced fatigue. There was no correlation between time from disease onset and subjective impression of recovery. Both groups exhibited impaired quality of life in cognitive and fatigue domains. SARS‐CoV‐2^+^ patients performed worse in attention and working memory cognitive tasks compared to a demographic‐matched US population (T‐score 41.5 [37, 48.25] and 43 [37.5, 48.75], respectively; both *p* < 0.01).

**Interpretation:**

Non‐hospitalized Covid‐19 “long haulers” experience prominent and persistent “brain fog” and fatigue that affect their cognition and quality of life.

## Introduction

As of 10 March 2021, severe acute respiratory syndrome coronavirus type 2 (SARS‐CoV‐2) has led to over 117 million confirmed infections and 2.6 million deaths from coronavirus disease‐2019 (Covid‐19) worldwide.^1^ Although SARS‐CoV‐2 manifests primarily with respiratory tract infections and flu‐like symptoms, Covid‐19 is now recognized as a multi‐organ disease often involving the nervous system.

Neurologic manifestations of varying severity^2^, ^3^, ^4^ have been reported in 36.4–82.3% of hospitalized Covid‐19 patients worldwide.^5^, ^6^, ^7^ Neurologic, pulmonary, cardiac, and gastrointestinal dysfunction may persist in the post‐acute phase and constitute a “long Covid” syndrome,^8^, ^9^ which has also recently been called the syndrome of “post‐acute sequelae of SARS‐CoV‐2 infection” (PASC).^10^ In addition, approximately 80% of infected individuals have limited and transient respiratory symptoms, and do not require hospitalization for pneumonia or hypoxemia.^11^, ^12^ Nevertheless, some develop persistent and debilitating symptoms despite a relatively mild illness at onset, and they are known as Covid‐19 “long haulers.”^4^, ^13^, ^14^

Whereas some “long haulers” were found to be positive for SARS‐CoV‐2 RNA by RT‐PCR at symptom onset, many did not fulfill the criteria for testing at the beginning of the pandemic, or tested negative at a time when respiratory symptoms had subsided. In addition, some “long haulers” did not have detectable antibodies to SARS‐CoV‐2 when the first serological test (Abbott) became available commercially. Whether this represents false negative results due to transient production of antiviral antibodies or the limited sensitivity of the assay is currently unclear.^15^, ^16^, ^17^

We sought to characterize the range of neurologic manifestations in non‐hospitalized “long haulers” presenting at our Neuro‐Covid‐19 clinic, in both SARS‐CoV‐2 laboratory‐positive (SARS‐CoV‐2^+^) and laboratory‐negative (SARS‐CoV‐2^‐^) individuals. Furthermore, cognitive dysfunction, identified as “brain fog” by “long haulers,” has been prominently mentioned in the media and in other studies.^8^, ^13^, ^18^, ^19^ Therefore, we prospectively evaluated multiple domains of cognitive function and self‐reported quality of life measures using validated instruments in Covid‐19 “long haulers”.

## Subjects/materials and methods

### Patients

We prospectively analyzed 100 patients (the first 50 consecutive SARS‐CoV‐2 laboratory‐positive [SARS‐CoV‐2^+^] and the first 50 consecutive SARS‐CoV‐2 laboratory‐negative [SARS‐CoV‐2^‐^] individuals who met study inclusion criteria) seen at the Neuro‐Covid‐19 clinic of Northwestern Memorial Hospital, Chicago, IL between 13 May and 11 November 2020. The first identified case of Covid‐19 in the United States was on 21 January 2020.^20^ Patients were included if they had clinical manifestations of Covid‐19 compatible with Infectious Diseases Society of America (IDSA) guidelines starting in February 2020 or later,^21^ but did not require hospitalization for pneumonia or hypoxemia, and had neurologic symptoms persisting at least 6 weeks from symptom onset. Covid‐19 diagnosis was confirmed by SARS‐CoV‐2 reverse transcription‐polymerase chain reaction (RT‐PCR) of nasopharyngeal swab and/or SARS‐CoV‐2 antibody testing in 50 SARS‐CoV‐2^+^ patients, whereas those tests showed negative results in 50 SARS‐Cov‐2^‐^ patients. SARS‐Cov‐2^‐^ patients meeting IDSA Covid‐19 symptom guidelines were included as a comparison group of patients with clinically suspected post‐acute viral syndrome. All laboratory, radiologic, and electrophysiologic assessments were performed as part of routine clinical care. This study was approved by our institutional review board (STU00212627).

### Procedures

All patients were evaluated by an attending neurologist (IJK) assisted by a neuroimmunology fellow (ELG), nurse practitioners, and neurology residents. Patients from the entire United States could be seen in televisits only from 13 May to 1 July, due to pandemic lockdown, and in a mix of tele and in‐person visits thereafter. Medical records from patients located outside Illinois (including 21 states) were obtained and reviewed. Patient‐reported quality of life in cognition and fatigue domains was assessed using the validated Patient Reported Outcome Measurement Information System (PROMIS) assessment.^22^, ^23^ Patients presenting in‐person had the opportunity to complete a cognitive function evaluation with the National Institutes of Health (NIH) Toolbox v2.1 instrument, including assessments of: processing speed (pattern comparison processing speed test); attention and executive memory (inhibitory control and attention test); executive function (dimensional change card sort test); and working memory (list sorting working memory test).^24^, ^25^, ^26^, ^27^ Both PROMIS and NIH Toolbox results are expressed as T‐scores adjusted for age, education, gender, and race/ethnicity with a score of 50 representing the normative mean/median of the US reference population with a standard deviation of 10. Lower cognition T‐scores indicate worse performance while higher fatigue T‐scores indicate greater fatigue severity.

### Statistical analysis

Data were summarized as number of patients (frequency), mean (standard deviation) for normally distributed variables, and median (interquartile range [IQR]) for non‐normally distributed variables. Group differences were assessed using Fisher’s exact test, unpaired t‐test, and Wilcoxon rank‐sum test. Correlations between variables were assessed with Pearson’s or Spearman’s correlation tests, as appropriate. To determine if results of PROMIS and NIH Toolbox domains differed from expected, patient group T‐scores were compared to the demographic‐matched normative US population median of 50 using one‐sample Wilcoxon signed‐rank tests. Two‐sided *p* ≤ 0.05 was considered significant and all analyses were performed in GraphPad Prism version 9.0.0. Study data were collected and managed using REDCap electronic data capture tools.

## Results

### Patient demographics, SARS‐CoV‐2 testing, and comorbidities

We cared for 135 patients in the Neuro‐Covid‐19 clinic between 13 May and 11 November 2020. Thirty‐five patients did not meet criteria for study inclusion: 20 had been hospitalized for pneumonia or hypoxemia, three had symptoms for less than 6 weeks, eight did not meet IDSA symptoms for Covid‐19, and four had symptom onset before 1 February 2020. As a result, the first 50 SARS‐CoV‐2^+^ and first 50 SARS‐CoV‐2^‐^ patients were included in this study.

Of the 100 patients included in this analysis, the mean age was 43.2 ± 11.3 years, 70% were female, and 88% were white. We saw 48 patients through televisits and 52 in‐person. Of the 50 SARS‐CoV‐2^+^ patients, 38 (76%) had a positive nasopharyngeal SARS‐CoV‐2 RT‐PCR and 28 (56%) had a positive SARS‐CoV‐2 serology. Of note, six (12%) had a negative nasopharyngeal SARS‐CoV‐2 RT‐PCR and four (8%) had a negative SARS‐CoV‐2 serology, and only 16 (32%) had both tests positive. The most common comorbidities prior to Covid‐19 diagnosis were depression/anxiety (42%), autoimmune disease (16%), insomnia (16%), lung disease (16%), and headache (14%). There were no significant demographic differences between the two groups, but the SARS‐CoV‐2^+^ subjects tended to have more frequent depression/anxiety prior to Covid‐19 than the SARS‐CoV‐2^‐^ subjects (26/50 (52%) vs. 16/50 (36%); p = 0.07). Patient demographics are summarized in Table 1.

**Table 1:** Study subjects’ demographics and comorbidities by SARS‐CoV‐2 result.

	Overall	SARS‐CoV‐2^+^	SARS‐CoV‐2^‐^	*p*
n	100	50	50	
Age, years (mean (1 SD))	43.2 (11.3)	43.7 (11.8)	42.6 (10.8)	0.62
Male, n (%)	30 (30)	17 (34)	13 (26)	0.51
Female, n (%)	70 (70)	33 (66)	37 (74)	
BMI (median [IQR])	25.4 [22.2‐30.1]	25.8 [23.6‐30.0]	24.7 [21.3‐30.2]	0.25
BMI > 25, n (%)	55 (55)	30 (60)	25 (50)	0.42
BMI > 30, n (%)	26 (26)	13 (26)	13 (26)	1
Race, n (%)				1
White	88 (88)	44 (88)	44 (88)	
Black or African American	6 (6)	2 (4)	4 (8)	
Asian	2 (2)	2 (4)	0 (0)	
American Indian or Alaskan Native	1 (1)	0 (0)	1 (2)	
Other	3 (3)	2 (4)	1 (2)	
Ethnicity, n (%)				1
Not Hispanic or Latino	88 (88)	44 (88)	44 (88)	
Hispanic or Latino	12 (12)	6 (12)	6 (12)	
Visit type, n (%)				0.32
In‐Person	52 (52)	29 (58)	23 (46)	
Televisit	48 (48)	21 (42)	27 (54)	
SARS‐CoV‐2 RT‐PCR, n (%)				<0.0001
Positive	38 (38)	38 (76)	0 (0)	
Negative	46 (46)	6 (12)	40 (80)	
Not performed	16 (16)	6 (12)	10 (20)	
SARS‐CoV‐2 Serology, n (%)				<0.0001
Positive	28 (28)	28 (56)	0 (0)	
Negative	48 (48)	4 (8)	44 (88)	
Not performed	24 (24)	18 (36)	6 (12)	
Positive RT‐PCR and serology, n (%)	16 (16)	16 (32)	0 (0)	<0.0001
Any preexisting comorbidity n (%)	42 (42)	22 (44)	20 (40)	0.84
Depression/anxiety	42 (42)	26 (52)	16 (36)	0.07
Autoimmunedisease^1^	16 (16)	7 (14)	9 (18)	0.79
Insomnia	16 (16)	10 (20)	6 (12)	0.25
Lungdisease^2^	16 (16)	9 (18)	7 (14)	0.79
Headache	14 (14)	5 (10)	9 (18)	0.39
Dyslipidemia	10 (10)	6 (12)	4 (8)	0.74
Cardiovasculardisease^3^	9 (9)	6 (12)	3 (6)	0.49
Traumaticbraininjury	8 (8)	3 (6)	5 (10)	0.72
Cancer	7 (7)	5 (10)	2 (4)	0.44
Dysautonomia	4 (4)	3 (6)	1 (2)	0.62
Type2Diabetes	2 (2)	1 (2)	1 (2)	1
Other^4^	17 (17)	9 (18)	8 (16)	1

^1^ Multiple sclerosis (1), systemic lupus erythematosus (3), Hashimoto’s thyroiditis (5), type 1 diabetes (1), psoriasis (1), celiac disease (2), eosinophilic esophagitis (1), ulcerative colitis (1), primary sclerosing cholangitis (1), Behcet’s disease (1), Raynaud’s (1), and rheumatoid arthritis (1). Three patients each had two autoimmune diseases.

^2^ Obstructive sleep apnea (9), asthma (5), and chronic obstructive pulmonary disease (2).

^3^ Stroke (1), hypertension (5), congestive heart failure (1), and atrial fibrillation (2).

^4^ Concern for “chronic Lyme” (2), secondary syphilis (1), Ehlers‐Danlos syndrome (1), fibromyalgia (3), attention‐deficit hyperactivity disorder (5), post‐traumatic stress disorder (2), narcolepsy (1), restless leg syndrome (1), and neurofibromatosis type 2 (1).

### Frequency of neurologic symptoms and signs attributed to Covid‐19

Patients were seen on average at 4.72 months after symptom onset in the SARS‐CoV‐2^+^ group compared to 5.82 months in the SARS‐CoV‐2^‐^ group (*p* = 0.002). Their subjective impression of recovery as compared to pre‐Covid‐19 baseline was 67.8% in the SARS‐CoV‐2^+^ group versus 60.3% in the SARS‐CoV‐2^‐^ group (*p* = 0.09).

Overall, patients reported a median of five neurologic symptoms related to Covid‐19, and 85% reported at least four symptoms, with no difference between the two groups. The 10 most frequent neurologic symptoms were non‐specific cognitive complaints, referred to as “brain fog” by patients (81%), headache (68%), numbness/tingling (60%), dysgeusia (59%), anosmia (55%), myalgia (55%), dizziness (47%), pain (43%), blurred vision (30%), and tinnitus (29%). Many patients reported fluctuating symptoms and most symptoms had not completely resolved by their clinic visit. For example, 33/55 (60%) patients still experienced some degree of anosmia. Only anosmia and blurred vision varied significantly between the two groups. SARS‐CoV‐2^+^ patients reported anosmia more frequently (37/50 [74%] vs. 18/50 [36%]; *p* < 0.001). Conversely, SARS‐CoV‐2^‐^ patients reported blurred vision more frequently (21/50 [42%] vs. 9/50 [18%], *p* = 0.02). The most frequent non‐neurologic symptoms included fatigue (85%), depression/anxiety (47%), shortness of breath (46%), chest pain (37%), insomnia (33%), variation of heart rate and blood pressure (30%), and gastrointestinal symptoms (29%), with no significant differences between the groups.

We performed a complete neurologic exam in the 52 patients who came to the clinic and a limited exam in the 48 televisit patients. Overall, 53% had an abnormal exam, and the most frequent neurologic signs were short‐term memory deficit by 4‐item recall (32%) and attention deficit by serial 7s (27%). The neurologic exam showed no significant difference between the two groups, but cranial nerve dysfunction tended to be more frequent in SARS‐CoV‐2^+^ than SARS‐CoV‐2^‐^ subjects (5/50 (10%) vs. 0/50 (0%); p = 0.06). The neurological manifestations are shown in Table 2.

**Table 2:** Neurologic symptoms and signs attributed to Covid‐19.

	Overall	SARS‐CoV‐2^+^	SARS‐CoV‐2^‐^	*p*
Time from onset (month, mean (1 SD))	5.27 (1.83)	4.72 (1.92)	5.82 (1.56)	0.002
Subjective impression of recovery compared to pre‐Covid‐19 baseline (mean % (1 SD))	63.9 (20.7)	67.8 (18.8)	60.3 (21.9)	0.09
Number of neurologic symptoms attributed to Covid‐19 (median [IQR])	5 [4‐7]	5 [4‐6]	5.5 [4‐7]	0.74
Neurologic symptom n (%)				
≥4	85 (85)	43 (86)	42 (84)	1
Brain fog	81 (81)	41 (82)	40 (80)	1
Headache	68 (68)	32 (64)	36 (72)	0.52
Numbness/tingling	60 (60)	29 (58)	31 (62)	0.84
Dysgeusia	59 (59)	32 (64)	27 (54)	0.42
Anosmia	55 (55)	37 (74)	18 (36)	<0.001
Myalgia	55 (55)	30 (60)	25 (50)	0.42
Dizziness	47 (47)	20 (40)	27 (54)	0.23
Pain other than chest	43 (43)	20 (40)	23 (46)	0.69
Blurred vision	30 (30)	9 (18)	21 (42)	0.02
Tinnitus	29 (29)	12 (24)	17 (34)	0.38
Movement disorder^1^	5 (5)	2 (4)	3 (6)	1
Focal motor deficit^2^	5 (5)	1 (2)	4 (8)	0.36
Focal sensory deficit	2 (2)	0 (0)	2 (4)	0.5
Dysarthria	2 (2)	2 (4)	0 (0)	0.5
Ataxia	1 (1)	0 (0)	1 (2)	1
Seizure	1 (1)	0 (0)	1 (2)	1
Dysphagia	1 (1)	1 (2)	0 (0)	1
Aphasia	1 (1)	1 (2)	0 (0)	1
Other symptom n (%)				
Fatigue	85 (85)	42 (84)	43 (86)	1
Depression/Anxiety	47 (47)	20 (40)	26 (52)	0.42
Shortness of breath	46 (46)	19 (38)	27 (54)	0.16
Chest pain	37 (37)	14 (28)	23 (46)	0.1
Insomnia	33 (33)	18 (36)	15 (30)	0.67
Variations in HR and BP^3^	30 (30)	9 (18)	21 (42)	0.02
GI symptoms^4^	29 (29)	14 (28)	15 (30)	0.38
Sign n (%)				
Abnormal exam	53 (53)	26 (52)	27 (54)	1
Short‐term memory deficit	32 (32)	15 (30)	17 (34)	0.83
Attention deficit	27 (27)	12 (24)	15 (30)	0.65
Sensory dysfunction^5^	8/52 (15.4)	3/29 (10.3)	5/23 (21.7)	0.44
Cranial nerve dysfunction^6^	5 (5)	5 (10)	0 (0)	0.06
Gait dysfunction	5 (5)	3 (6)	2 (4)	1
Motor dysfunction	4 (4)	3 (6)	1 (2)	0.62
Movement disorder	2 (2)	0 (0)	2 (4)	0.49
Cerebellar dysfunction	1 (1)	1 (2)	0 (0)	1

^1^ Self‐reported abnormal movements (5).

^2^ Hand weakness lasting days to weeks—right‐sided (3), left‐sided (1). The one patient in the SARS‐CoV‐2^+^ group was found to have an acute medullary infarct, whereas work‐up in the three SARS‐CoV‐2^‐^ patients was unrevealing.

^3^ Self‐reported rapid variations of heart rate (HR) (24), blood pressure (BP) (6), and unspecified (3).

^4^ Diarrhea (19), nausea (12), vomiting (2), and gastroparesis (2).

^5^ Evaluated for in‐person visits only.

^6^ Decreased hearing (2), gaze‐evoked nystagmus (2), and facial droop (1).

### Radiological, electrophysiological, and laboratory testing, and medications trialed

Diagnostic testing done prior to and at the time of visit is listed in Table 3 and showed no differences between the two groups. Markers of inflammation evaluated included antinuclear antibody (ANA), with a titer >1:160 found in 3/6 (50%) SARS‐CoV‐2^+^ versus 8/27 (29%) SARS‐CoV‐2^‐^ patients tested (*p* = 0.38). Of the patients with ANA titer >1:160, 1/3 SARS‐CoV‐2^+^ and 1/8 SARS‐CoV‐2^‐^ had preexisting autoimmune disease. Erythrocyte sedimentation rate, C‐reactive protein, D‐dimer, and ferritin checked at any point since symptom onset were not significantly different between the two groups. Medications trialed for Covid‐19‐related symptoms, either before or at the time of the office visit, varied. Many patients were either previously on or started antidepressants (31%), benzodiazepines (19%), or gabapentin (11%). Other medications prescribed included amantadine (6%), valacyclovir (3%), prednisone (3%), dexamethasone (2%), hydroxychloroquine (2%), modafinil (2%), and colchicine (1%) with no differences between the two groups.

**Table 3:** Diagnostic testing.

	Overall	SARS‐CoV‐2^+^	SARS‐CoV‐2^‐^	*p*
n abnormal/n tested (%)				
Brain MRI^1^	9/48 (18.8)	5/22 (22.7)	4/26 (15.4)	0.71
MR Vessel Wall Imaging	0/4 (0)	0/2 (0)	0/2 (0)	1
Spine MRI^2^	10/16 (62.5)	5/8 (62.5)	5/8 (62.5)	1
EMG^3^	3/9 (33)	1/3 (33)	2/6 (33)	1
EEG	0/4 (0)	0/3 (0)	0/1 (0)	1
CSF analysis ^4^	3/5 (60)	0/1 (0)	3/4 (75)	0.40
Tilt table test	3/4 (75)	0 (0)	3/4 (75)	1
Antinuclear antibody ≥ 1:160	11/33 (33.3)	3/6 (50)	8/27 (29.6)	0.38
Erythrocyte sedimentation rate				
Median [IQR], Reference: Males: <15 (0‐50 years) or < 20 mm/h (51‐85 years). Females: <20 (0‐50 years) or < 30 mm/h, (51‐85 years).	8/47 (17) 9 [3‐19]	2/15 (13.3) 11 [2‐19]	6/32 (18.8) 8.5 [3.75‐19.5]	1
C‐reactive protein				
Median [IQR], Reference: 0.0‐0.5 mg/dL	10/52 (19.2) 0.5 [0.29‐0.57]	5/19 (26.3) 0.5 [0.5‐1.2]	5/33 (15.2) 0.4 [0.24‐0.5]	0.47
D‐dimer				
Median [IQR], Reference: 0‐230 ng/mL	8/27 (29.6) 174.5 [150‐329]	3/10 (30) 150 [150‐289]	5/17 (29.4) 215 [163‐327]	1
Ferritin				
Median [IQR], Reference: 24‐336 ng/mL	2/11 (18.2) 75 [42‐120]	2/5 (40) 105 [42‐120]	0/6 (0) 65.2 [50.7‐88.5]	0.18

^1^ Non‐specific white matter changes (5), microhemorrhage (1), infarction (1), demyelinating disease (1), and schwannoma (1).

^2^ Degenerative changes (9) and thecal sac diverticula vs. perineural cysts (1).

^3^ Axonal sensory‐predominant polyneuropathy prior to Covid‐19 onset (1), absent medial plantar SNAP (1) due to orthopedic injury, possible S1 radiculopathy (1).

^4^ Pleocytosis (1) [12 WBC, 100% lymphocytes], elevated protein (3) (reference) [52 (15‐45), 56 (15‐45), 49 (0‐35)].

### Quality of life measures and standardized cognitive tests

**Figure 1:**
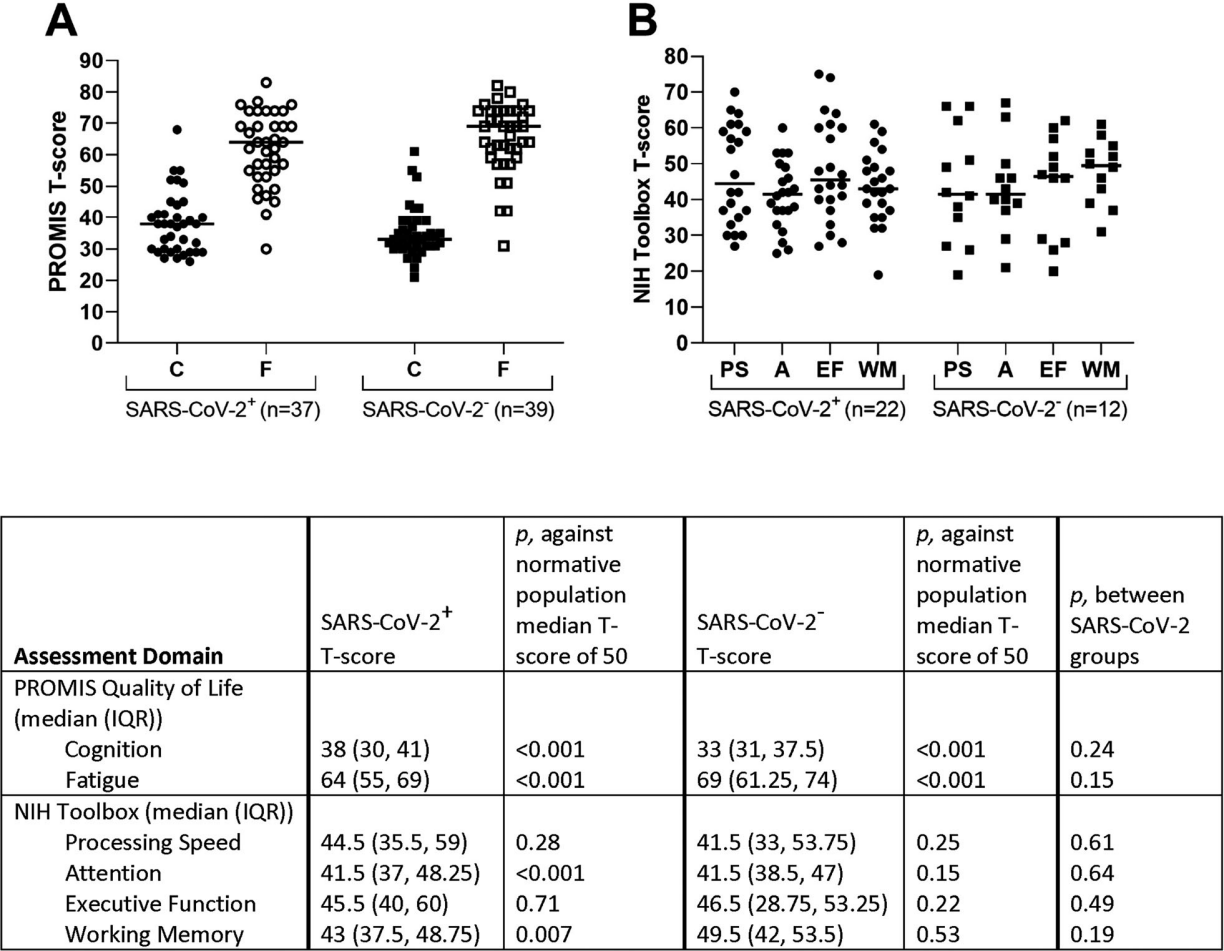
Patient‐reported outcomes measurement information system (PROMIS®) quality of life and NIH Toolbox cognitive assessments demographic‐matched T‐scores in SARS‐CoV‐2^+^ (circles) and SARS‐CoV‐2^‐^ (squares) individuals. A T‐score of 50 is the mean/median for the demographic‐matched United States normative population with a standard deviation of 10. (A) PROMIS® cognitive function (C, filled symbols) and fatigue (F, empty symbols) assessments. Lower cognition scores indicate worse cognition quality of life and higher fatigue scores correspond to worse fatigue quality of life. Patient group median values are represented by horizontal bars. (B) NIH Toolbox assessments for processing speed (PS), attention (A), executive function (EF), and working memory (WM). Median values are represented by horizontal bars. One‐sample Wilcoxon signed‐rank test p‐values between patient group T‐scores and the demographic‐matched normative US population median of 50 are provided in the figure table.

The results of PROMIS quality of life and NIH Toolbox cognition assessments are shown in Figure 1, reported as T‐scores accounting for patients’ age, level of education, race/ethnicity, and gender. The PROMIS inventory was completed by 76% of patients (37 SARS‐CoV‐2^+^ and 39 SARS‐CoV‐2^‐^). Thirty‐six percent of the cohort (24/50 (48%) SARS‐CoV‐2^+^ and 12/50 (24%) SARS‐CoV‐2^‐^, 69.2% of the in‐person visits) underwent cognitive assessment with NIH Toolbox. Toolbox T‐scores were unavailable for two SARS‐CoV‐2^+^ patients due to baseline education level or racial/ethnic backgrounds, thus 22 SARS‐CoV‐2^+^ and 12 SARS‐CoV‐2^‐^ T‐scores were analyzed. PROMIS and NIH Toolbox results were not significantly different between SARS‐CoV‐2^+^ and SARS‐CoV‐2^‐^ groups, with the median T‐scores indicating moderate cognition and fatigue quality of life impairment and mild‐to‐moderate cognitive dysfunction for both groups (Figure 1). However, compared to the demographic‐matched US normative population, both SARS‐CoV‐2^+^ and SARS‐CoV‐2^‐^ patients had significantly worse than expected PROMIS quality of life for cognition (median T‐score 38 [30‐41] and 33 [31‐37.5], respectively; both *p* < 0.001 vs. US median of 50) and fatigue (median T‐score 64 [55, 69] and 69 [61.25, 74], respectively; both *p* < 0.001 vs. US median of 50). Furthermore, compared to the expected demographic‐matched US normative population, SARS‐CoV‐2^+^ patients had significantly worse NIH Toolbox cognitive function in attention (median T‐score 41.5 [37, 48.25]; *p* < 0.001 vs. US median of 50) and working memory (median T‐score 43 [37.5, 48.75]; *p* = 0.007 vs. US median of 50) domains. While SARS‐CoV‐2^‐^ patients tended to have worse NIH Toolbox performance than the expected US normative population, this did not achieve statistical significance in any cognitive domain (*p* ≥ 0.15 for all domains vs. US median of 50).

Nineteen SARS‐CoV‐2^+^ and nine SARS‐CoV‐2^‐^ patients completed both PROMIS and NIH Toolbox assessments. Using Spearman’s correlations and the entire patient cohort, PROMIS fatigue quality of life T‐scores were moderately correlated with NIH Toolbox T‐scores for processing speed (r = −0.45, *p* = 0.02), executive function (r = −0.43, *p* = 0.02), and working memory (r = −0.44, *p* = 0.02); meanwhile, PROMIS cognition quality of life T‐scores were only correlated with NIH Toolbox T‐scores for working memory (r = −0.44, *p* = 0.02). Correlation coefficients between PROMIS and NIH toolbox domains were similar for both groups, except that SARS‐CoV‐2^‐^ patients demonstrated a strong inverse correlation between fatigue severity and attention function (r = −0.76, *p* = 0.02), while SARS‐CoV‐2^+^ patients demonstrated no correlation between fatigue severity and attention function (r = −0.07, *p* = 0.79).

### Assessment of recovery to pre‐Covid‐19 baseline

**Figure 2:**
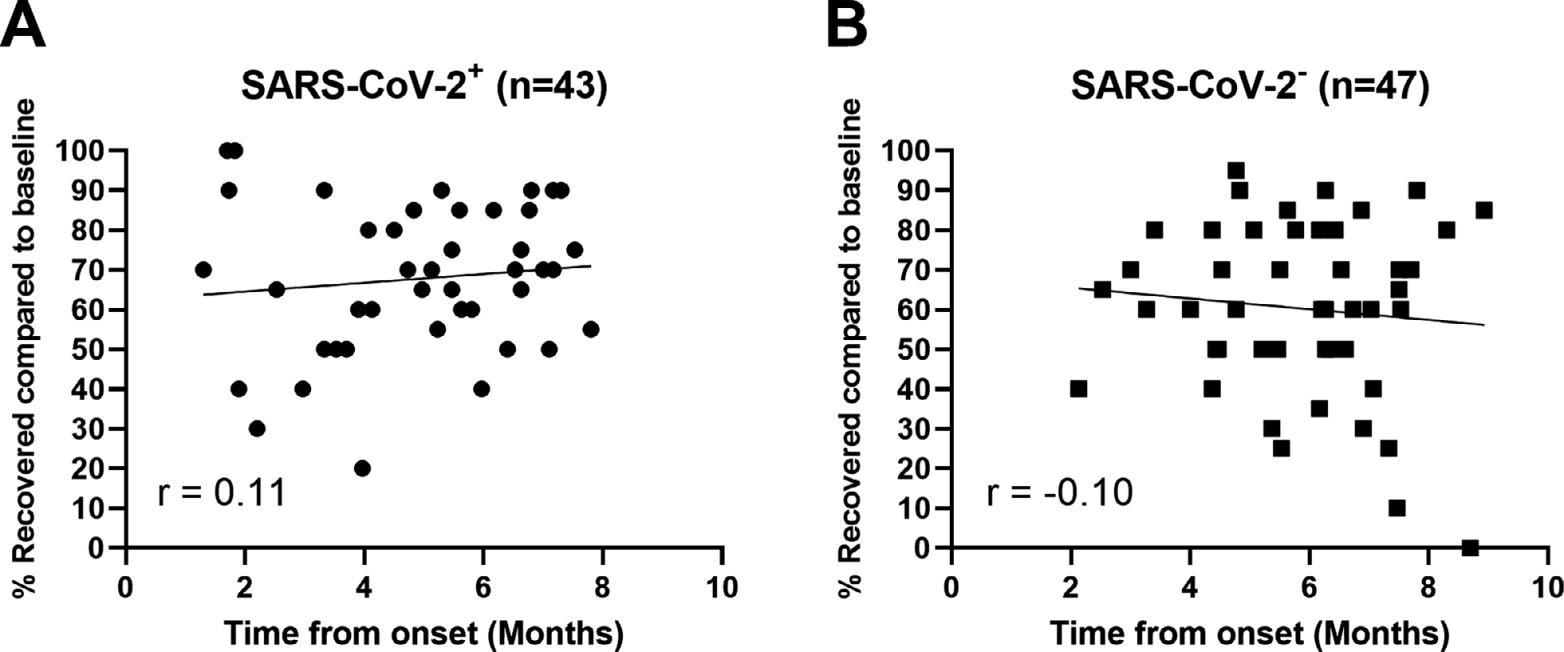
Subjective impression of recovery compared to pre‐Covid‐19 baseline for SARS‐CoV‐2^+^ (A) and SARS‐CoV‐2^‐^ individuals (B). The patients were asked to grade their recovery at the time of their visit, assuming a pre‐Covid‐19 baseline of 100%. Each person is represented by a single time point, and r values demonstrate no meaningful relationship between time from onset and percentage of recovery.

Time from symptom onset was not associated with subjective impression of recovery compared to pre‐Covid‐19 baseline (Figure 2; SARS‐CoV‐2^+^ Pearson’s r = 0.11, *p* = 0.49, SARS‐CoV‐2^‐^ Pearson’s r = −0.10, *p* = 0.51). However, patients’ subjective report of recovery toward pre‐Covid‐19 baseline was moderately correlated with PROMIS fatigue (Spearman’s r = −0.40, *p* < 0.001) and cognition (Spearman’s r = 0.45, *p* < 0.001) T‐scores but was not correlated with any NIH Toolbox domain. Lastly, SARS‐CoV‐2^‐^ patients were more likely to miss over 10 days of work due to their residual symptoms compared to SARS‐CoV‐2^+^ patients (27/46 (59%) vs. 16/44 (36%); *p* = 0.04).

## Discussion

### Definition of long Covid‐19 in non‐hospitalized individuals

This study characterizes the broad range of neurologic manifestations in SARS‐CoV‐2^+^ and SARS‐CoV‐2^‐^ “long haulers” seeking care at our Neuro‐Covid‐19 clinic. We opened this clinic in May 2020 in response to the high frequency of nervous system dysfunction recognized in hospitalized Covid‐19 patients in China and Europe, as well as in our own in‐patient population.^5^, ^6^, ^7^ Despite the high frequency of neurologic involvement in 82% of hospitalized Covid‐19 patients,^7^ the large majority of our Neuro‐Covid‐19 clinic population consisted of individuals who were never hospitalized for respiratory complications of Covid‐19. All patients in this study had clinical symptoms consistent with Covid‐19 by IDSA criteria, but only had mild and transient respiratory symptoms, and never developed pneumonia or hypoxemia requiring hospitalization. The definition of long Covid‐19 is not settled. For the purposes of this study, we defined long Covid‐19, also known as PASC,^10^ as symptoms lasting more than 6 weeks given population surveys indicating a substantial majority of patients fully recovered by 4‐6 weeks.^28^, ^29^, ^30^

### Rationale for inclusion of SARS‐CoV‐2^‐^ patients as control group with post‐acute viral syndrome

The concept that SARS‐CoV‐2^‐^ individuals could in fact have Covid‐19 is contrary to the common belief that diagnosis of a viral disease requires detection of viral nucleic acids or proteins at the site of infection, or proof of a humoral immune response to the virus. However, this assumption is entirely dependent on the type of viral infection and sensitivity of the assays. SARS‐CoV‐2^‐^ patients were included by design in this study given the absence of a gold standard for diagnostic testing for Covid‐19 and to serve as an internal post‐acute viral syndrome control group. Detection of SARS‐CoV‐2 RNA in nasopharyngeal swab is dependent on viral shedding, which may not occur in individuals without persistent respiratory symptoms.^31^ In addition, the first serological test that became available to most of our study subjects measured antibodies against SARS‐CoV‐2 nucleocapsid (Abbott). This assay was developed under emergency use authorization using a limited number of blood samples from hospitalized patients with pneumonia.^32^ It was therefore not designed to detect SARS‐CoV‐2 antibodies in non‐hospitalized “long haulers”.^15^, ^16^, ^33^ Furthermore, production of antibodies against SARS‐CoV‐2 may be time‐limited, and may become undetectable after only weeks to months.^34^, ^35^, ^36^, ^37^, ^38^, ^39^ This could explain why SARS‐CoV‐2^‐^ “long haulers” who were infected with the virus early in the pandemic remain seronegative even with the more sensitive assays detecting antibodies against the virus spike protein, which only became commercially available recently.^17^, ^40^

Lack of antibody detection despite evidence of active infection has previously been well demonstrated with other viruses, including hepatitis C virus and JC polyomavirus.^41^, ^42^ Hence, it is not possible to determine with certainty that the SARS‐CoV‐2^‐^ individuals in this study have not been exposed to the coronavirus. Nevertheless, those patients comprised half of our clinic population during the period under investigation and therefore constitute the most closely matched control group with a clinically suspected post‐acute viral syndrome for comparison with the SARS‐CoV‐2^+^ patients, as recommended in the recent NIH PASC research opportunity announcement.^43^ It is particularly noteworthy that the demographics and comorbidities of our SARS‐CoV‐2^+^ and SARS‐CoV‐2^‐^ patients are quite similar. For example, both groups are approximately 15 years younger than the hospitalized Covid‐19 patients at our institution.^7^ Due to the shortcomings of using antibodies to establish the diagnosis of Covid‐19 in non‐hospitalized patients, we are now studying the T‐cell response of “long haulers” against SARS‐CoV‐2 proteins as a potential means to improve the identification of patients who were in fact infected with SARS‐CoV‐2.

### Increased female:male ratio and comorbidities suggest autoimmune etiology of long Covid‐19

While our study was not designed to identify the mechanisms underlying the “long hauler” phenomena, several features of our cohort suggest potential contributors. The female:male ratio of 2.3:1 is reminiscent of autoimmune diseases such as multiple sclerosis: 2:1,^44^ rheumatoid arthritis: 3:1,^45^ and systemic lupus erythematosus: 7:1.^46^ The prevalence of preexisting autoimmune disease and elevated ANA titer >1:160 in our cohort of “long haulers” compared to the general population (16% vs. 7% and 33% of those tested vs. 5%, respectively)^47^, ^48^ suggests the possibility of an autoimmune contribution. Moreover, contributions from hypoxemia or chronic vascular disease seem unlikely to explain the “long hauler” phenomena given that our cohort did not experience respiratory symptoms requiring hospitalization and had lower rates of comorbid cardiovascular disease, diabetes, and hyperlipidemia than are reported in severe Covid‐19.^49^, ^50^, ^51^ Premorbid depression/anxiety was also prevalent in our cohort (42% vs. 21.4% of US adults with mood disorder),^52^ suggesting a possible neuropsychiatric vulnerability to becoming a “long hauler” after SARS‐CoV‐2 infection.^53^

Finally, other mechanisms may also be contributing to neurologic symptoms of long Covid, including infection or inflammation of endothelial cells of brain vessels (endotheliitis). Indeed, the recent observation of megakaryocytes in cortical capillaries of the brain from patients who died from Covid‐19 suggests that a possible microvasculopathy or perhaps the release of chemically active substances, such as serotonin, from these megakaryocytes might play a role in “long hauler” symptoms.^54^ We have also used transcranial Doppler to demonstrate intracerebral microemboli in hospitalized Covid‐19 patients; these microemboli could also contribute to Covid‐19‐associated encephalopathy through capillary occlusion.^55^ Further research is needed to determine whether endotheliitis or a microvasculopathy contribute to the neurologic manifestations occurring in non‐hospitalized “long haulers”.

### Multiplicity of neurologic symptoms and cognitive dysfunction impact quality of life

By history, fatigue was the single most reported symptom and 85% of patients experienced four or more neurologic symptoms, with the most frequent being “brain fog”—the colloquial term used by “long haulers” to describe their lingering cognitive difficulties. The high rate of encephalopathy in hospitalized Covid‐19 patients leads one to question whether “brain fog,” with or without fatigue, might represent a mild form of post‐Covid‐19 encephalopathy.^7^ Approximately half of the patients in our study had an abnormal neurologic exam, with abnormalities on short‐term memory and attention functions being prominent. Consistent with these history and exam findings, we found that both SARS‐CoV‐2^+^ and SARS‐CoV‐2^‐^ “long haulers” had significantly worse quality of life in the areas of cognition and fatigue than would be expected based on their demographic features, and the median cognition and fatigue quality of life impairment were of moderate severity.^56^ Furthermore, NIH Toolbox cognitive assessments identified that SARS‐CoV‐2^+^ patients had significantly worse attention and working memory function than would be expected based on their demographic features. Limited sample size may have prevented us from detecting cognitive dysfunction, in domains such as attention, in the SARS‐CoV‐2^‐^ “long haulers”; alternatively, the presence of objective impairment in certain cognitive domains, such as attention or working memory, might be a feature that could distinguish “long hauler” groups and should be investigated in future, larger studies.

Interestingly, “long haulers’” fatigue‐based quality of life was more clearly correlated with NIH Toolbox cognitive function than was cognition‐based quality of life. Additionally, the relationship between fatigue severity and attention might distinguish “long hauler” groups, with only SARS‐CoV‐2^‐^ patients demonstrating significantly worse attention with increasing fatigue. These data suggest that “long haulers” might have better insight into their fatigue than cognitive quality of life, and that insight might differ between groups. These observations also raise the possibility that fatigue contributes to cognitive dysfunction in “long haulers” or that symptoms such as fatigue, depression, or anxiety might influence patients’ perception or experience of their cognitive function. Therefore, we are now studying the role of anxiety and depression, as well as quality of sleep, to determine their contribution to “brain fog” and fatigue of non‐hospitalized “long haulers”.

### Similarities of symptoms and stigma with myalgic encephalomyelitis/chronic fatigue syndrome (ME/CFS)

The constellation of “long hauler” symptoms, particularly fatigue and a sense of cognitive dysfunction, present in our “long hauler” patients resemble the prominent fatigue and cognitive complaints seen in those after mild traumatic brain injury, and in patients with myalgic encephalomyelitis/chronic fatigue syndrome (ME/CFS).^57^, ^58^, ^59^, ^60^, ^61^ Interestingly, SARS‐CoV‐2^‐^ patients sought Neuro‐Covid‐19 clinic consultation on average 1 month later after symptom onset than SARS‐CoV‐2^+^ patients. This may have been caused by the difficulty of SARS‐CoV‐2^‐^ individuals to find medical providers, since they elude classical molecular and serologic diagnostic criteria of Covid‐19.^62^, ^63^, ^64^ However, the anguish experienced by those patients who are suffering from multiple debilitating symptoms consistent with Covid‐19 but with no definitive diagnosis of SARS‐CoV‐2 infection should not be underestimated. Since the majority of our clinic patients are women, this is reminiscent of the stigma experienced by women with fibromyalgia and CFS.^65^ This potential stigma further highlights the need for improved diagnostic “gold standards” for SARS‐CoV‐2 infection, which our group hopes to address by elucidating the T‐cell response of “long haulers” against SARS‐CoV‐2 proteins.

### Study limitations

Our study has limitations in addition to the limited sample size. The large majority of patients were white, which precludes generalization to minority populations; however, televisits were offered to facilitate access to a broader range of patients. Approximately half of the patients were seen through televisits, which prevented features of the neurologic exam and NIH Toolbox assessment. Nevertheless, this also allowed us to include a more generalized representation of peoples from 21 states. We also did not have pre‐Covid‐19 quality of life or cognitive assessments, which prevented us from measuring the magnitude of change in individual patients; however, this is a limitation to be faced by any study addressing an acute, unpredictable medical condition, such as Covid‐19. We attempted to mitigate this limitation by using demographic‐adjusted T‐scores to compare groups to expected quality of life and cognitive function, as recommended by the National Institutes of Health.^66^ We also queried patients on their subjective return to premorbid baseline, which we found was significantly and moderately correlated with objective quality of life measures. Additionally, patients had a single evaluation and presented at different times from disease onset; therefore, our study was not designed to evaluate the evolution or fluctuation of symptoms over time. However, patients’ subjective report of percent recovery suggests that time after Covid‐19 may not be a good predictor of improvement toward baseline and that each individual may have a different recovery trajectory. Since testing was ordered based on clinical indication, not every patient had the same set of laboratory, imaging, and neurophysiologic testing; this could obscure potential relationships between phenomena such as autonomic dysfunction and “long hauler” symptoms. Similarly to any study performed in a clinic setting, our cohort also consists of a group of self‐selected individuals who sought evaluation in our Neuro‐Covid‐19 clinic. It is therefore representative of a specialized outpatient clinic population and should not be generalized to all non‐hospitalized SARS‐CoV‐2‐infected individuals. However, it allowed us to characterize precisely the many neurologic symptoms that have been commonly reported in population surveys.^67^ Finally, our group of SARS‐CoV‐2^‐^ patients may have been heterogeneous, including those who had really been infected by SARS‐CoV‐2 or its variants and others who could represent post‐viral syndromes caused by other viruses. In line with the early report of neurologic complications of HIV infection,^68^ our study highlights the need for future research and indicates that multiple distressing neurologic manifestations may occur and persist in the post‐acute phase of Covid‐19, even in patients who never needed hospitalization for pneumonia or hypoxemia.

### Frequency of long Covid‐19 in non‐hospitalized individuals and implications for future research

Our study indicates that long Covid‐19 is an important emerging entity requiring multidisciplinary expertise and care. It is estimated that 87% of hospitalized Covid‐19 patients continue to have symptoms 60 days after disease onset,^4^ and app‐based symptom trackers estimate that 4.5% of patients have mild Covid‐19 symptoms lasting greater than 8 weeks.^69^ Other studies report that half of non‐hospitalized Covid‐19 patients experienced at least one persisting symptom after a mean of 4 months.^70^ Accordingly, several million people in the world may already suffer from “long Covid.”

Further studies are needed to elucidate the pathogenesis of SARS‐CoV‐2 in the nervous system. Whereas hypoxemia, systemic inflammation, coagulopathy, and neuroinvasion have been implicated in hospitalized Covid‐19 patients who develop encephalopathy,^3^ it appears more likely that post‐infectious, autoimmune mechanisms may be at play in “long Covid.” The long‐term impact of “long Covid” on quality of life and potential return to normalcy, through lost productivity and lingering cognitive dysfunction, may be substantial as the pandemic continues to escalate. Future longitudinal studies are needed to evaluate the cognitive effect of SARS‐CoV‐2 infection on non‐hospitalized individuals, as they comprise the majority of Covid‐19 patients and may significantly impact workforce productivity.

## Conflict of Interest

The authors report no conflict of interest pertaining to this publication.

## Funding Information

No funding information provided.
